# Pretreatment FDG PET in prognosis of locoregionally advanced nasopharyngeal carcinoma treated with intensity-modulated radiation therapy

**DOI:** 10.7150/ijms.105995

**Published:** 2025-01-27

**Authors:** Xiaoshuang Niu, Fen Xue, Dan Ou, Yuming Zheng, Chaosu Hu, Chunying Shen, Xiayun He

**Affiliations:** 1 Department of Radiation Oncology, Fudan University Shanghai Cancer Center, Shanghai, China.; 2 Department of Oncology, Shanghai Medical College, Fudan University, Shanghai, China; Department of Radiation Oncology, Shanghai Clinical Research Center for Radiation Oncology, Shanghai, China; Shanghai Key Laboratory of Radiation Oncology, Shanghai, China.

## Abstract

**Objectives:** We aimed to investigate the long-term survival benefit of PET/CT compared with the routine examination (chest CT, abdominal enhanced CT and emission computed tomography (ECT)) for locally advanced nasopharyngeal carcinoma (NPC) before treatment.

**Methods:** From June 8^th^ 2005 to August 10^th^ 2017, 507 histologically diagnosed NPC patients with the 8th AJCC/UICC staging criteria III-IVA were enrolled in this study. Among them, patients underwent chest CT, abdominal enhanced CT and bone emission CT (control group), or replaced by positron emission tomography-CT (PET-CT group) to check for distant metastases.

**Results:** The numbers of patients in the control and PET-CT group were 344 (67.9%) and 163 (32.1%), respectively. With the median follow-up of 72 months, a total of 127 (25.0%) patients died. The 5-year and 8-year overall survival (OS) rates of the control and PET-CT group were 81.1% and 86.9%, 70.8% and 74.6% (P=0.087), respectively. Patients with T1-3, III stage and TPF showed improved 5-year and 8-year OS rates compared with T4, IVA stage and PF patients (P=0.001, P=0.000 and P=0.009). Patients with initially PET-CT-based staged showed improved 5-year and 8-year distant control (DC) compared with the control group (90.6% vs. 83.3% and 90.6% vs. 81.0%, P=0.013). There was no significant difference in local control (LC) and regional control (RC), between the control and PET-CT group.

**Conclusions:** Patients with initially PET-CT-based staged showed improved long-term DC compared with the control group. Initially PET-CT-based staged is recommended routinely in locoregionally advanced NPC.

## Introduction

The proportion of distant metastasis in nasopharyngeal carcinoma (NPC) at initial diagnosis is high, ranging from 9.9% to 14.8% 1-3. The common sites of distant metastasis are bone, liver and lung 4-5. The treatment principles and prognosis differ greatly with/without distant metastasis in NPC. The 5-year overall survival (OS) rate for patients without distant metastasis was between 77.8% and 84.0% 6-9, while the 2-year OS was only 50.2%-76.4% for distant metastasis patients at initial diagnosis 10-13.

Traditionally, in order to rule out distant metastasis for newly diagnosed NPC, chest X-ray, abdominal ultrasound and ECT were performed 14-16. PET-CT could detect potential distant metastasis, especially in medium-high risk patients 2, 16-20, while it might not be helpful in early-stage patients 21-22. Yen TC's 17 study included 118 cases of newly diagnosed NPC (N0: 16, N1: 31, N2: 53, N3: 18 cases) and 22 cases of recurrent NPC. Among the M0 patients after routine examination (chest radiography, whole-body bone scanning, and liver sonography), PET-CT could detect metastatic lesions in 12.9% cases. Xiao BB's 21 retrospective analysis showed that there were no differences in OS, progression-free survival (PFS), relapse-free survival (RFS) or distant metastasis-free survival (DMFS) between conventional workup (CWU: chest radiograph, liver ultrasound, bone scintigraphy) and PET-CT+CWU group for stage I-II NPC. In another retrospective analysis, Yang PC 22 included stage I-IVA NPC with/without PET-CT before treatment. All patients in the control group (non-initially PET-CT-based staged) received abdominal ultrasound and chest X-ray for metastatic staging. Propensity score matching yielded 4366 patients in each group. The 5-year OS rates were 76.3% and 71.2% (P=0.0015). The 5-year OS rates for stage I were 91.9% and 88.9% (P=0.4431); for stage II, III, and IV, they were 88.7% and 84.1% (P=0.0260), 82.4% and 77.6% (P=0.0104), 61.9% and 56.8% (P=0.0299), respectively. In conclusion, most routine examination before treatment was based on chest X-ray, abdominal ultrasound and ECT, with relatively short observation time. It is uncertain that whether PET-CT plays an important role in the treatment and prognosis of NPC. Therefore, we reported the 8-year efficacy of locally advanced NPC performed with control group (chest CT, enhanced abdominal CT and ECT), compared to the PET-CT group.

## Materials and Methods

### Patients

From June 8^th^ 2005 to August 10^th^ 2017, 507 histologically diagnosed (WHO II/III) NPC patients with the 8th AJCC/UICC staging III-IVA were enrolled in this study. All patients had no history of a second malignant tumor with KPS≥70 and provided informed written consent before treatment. Initial assessment consisted of enhanced magnetic resonance imaging (MRI) of the nasopharynx and enhanced MRI/CT of the neck, chest CT, abdominal enhanced CT and bone emission CT (control group), or replaced by positron emission tomography-CT (PET-CT group). All patients were re-staged according to the 8th AJCC/UICC staging criteria.

### Radiotherapy and chemotherapy

All patients were treated with IMRT. The prescribed dose given to primary tumor was 66 Gy in 30 fractions for T1 or T2 lesion and 70.4 Gy in 32 fractions for T3 or T4 disease (PTV-NX: GTV-NX +5 mm). A total dose of 66 Gy was given to the planned target volume of the lymph nodes (PTV-LN: GTV-LN +3 mm) in 30-32 fractions. The PTV-60 covering the high-risk CTV and a 5-mm margin was prescribed 60 Gy/30-32 F. The PTV-54 covering the low-risk CTV and a 5-mm margin was prescribed 54 Gy/30-32 F. Radiotherapy was given once daily, 5 fractions per week.

All patients received chemotherapy, including induction chemotherapy (IC) + concurrent chemotherapy (CCRT) or adjuvant chemotherapy (AC). Generally, the IC/AC regimens were delivered: TPF (docetaxel 60 mg/m2 d1, cisplatin 25 mg/m2 d1-3 and 5-FU 500 mg/m2 /d with 120-h infusion), or PF (cisplatin 25 mg/m2 d1-3 and 5-FU 500 mg/m2 /d with 120-h infusion). The regimens were repeated every 21 days for two cycles. CCRT was cisplatin 30 mg/m2 weekly or 80 mg/m2 d1-3 every 21 days during IMRT.

### Follow-up and statistical analysis

After treatment completion, follow-ups occurred every 3 months for the first 2 years, every 6 months from the third through the fifth year and annually thereafter.

SPSS 23.0 (SPSS Inc, Chicago, IL, USA) was used for statistical analysis in this study. The estimated OS, local control (LC), regional control (RC), and distant control (DC) were calculated by the Kaplan-Meier method. The duration of survival was measured from the time of treatment until death or the date of the last follow up visit for patients alive. LC was calculated from the date of initiation of treatment to the date of local failure or last follow-up. RC was calculated from the date of initiation of treatment to the date of regional failure or last follow-up. DC was calculated from the date of initiation of treatment to the date of metastasis or last follow-up. A 2-sided P<0.05 was considered statistically significant.

## Results

### Patient characteristics

From June 8^th^ 2005 to August 10^th^ 2017, 507 histologically diagnosed (WHO II/III) NPC patients with the 8th AJCC/UICC staging III-IVA were enrolled in this study. The numbers of patients in the control group and PET-CT group were 344 (67.9%) and 163 (32.1%), respectively. The median age was 48 years (range, 17-73 years). The study included 363 male (71.6%) and 144 female (28.4%). The treatment of patients with IC+RT+AC and IC+CCRT were 392 (77.3%) and 115 (22.7%), respectively. The characteristics of the two group patients were shown in Table 1.

### Overall survival

**Figure 1:**
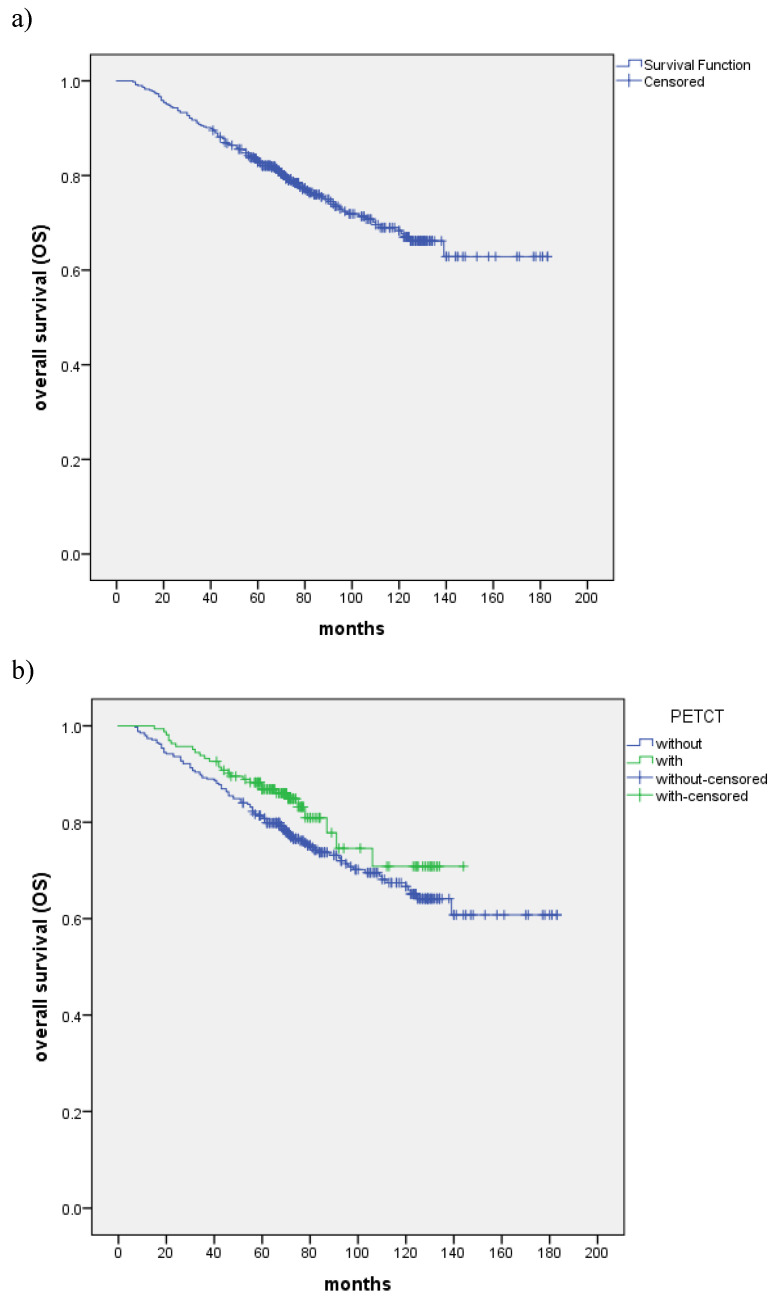
a) Kaplan-Meier estimate of OS curve for all the patients. b) The OS between patients with the control and PET-CT group, respectively.

With the median follow-up of 72 months, a total of 127 (25.0%) patients died: 32 patients died of recurrence in nasopharynx and/or regional lymph nodes, 7 of recurrence in regional lymph nodes, 46 patients died of distant metastasis, 8 of distant metastases accompanied by recurrence in nasopharynx and/or regional lymph nodes, 8 of distant metastases accompanied by recurrence in regional lymph nodes, 1 of massive hemorrhage, 6 of second tumor and 19 of other reasons. The all-cause death of the two group patients was shown in Table 2. The 5-year and 8-year OS rates were 82.9% and 72.4%, respectively (Fig. 1a). The 5-year and 8-year OS rates of the control and PET-CT group were 81.1% and 86.9%, 70.8% and 74.6% (P=0.087), respectively (Fig. 1b). Patients with T1-3, III stage and TPF showed improved 5-year and 8-year OS rates compared with T4, IVA stage and PF patients (P=0.001, P=0.000 and P=0.009) (Table 3).

### Distant control

**Figure 2:**
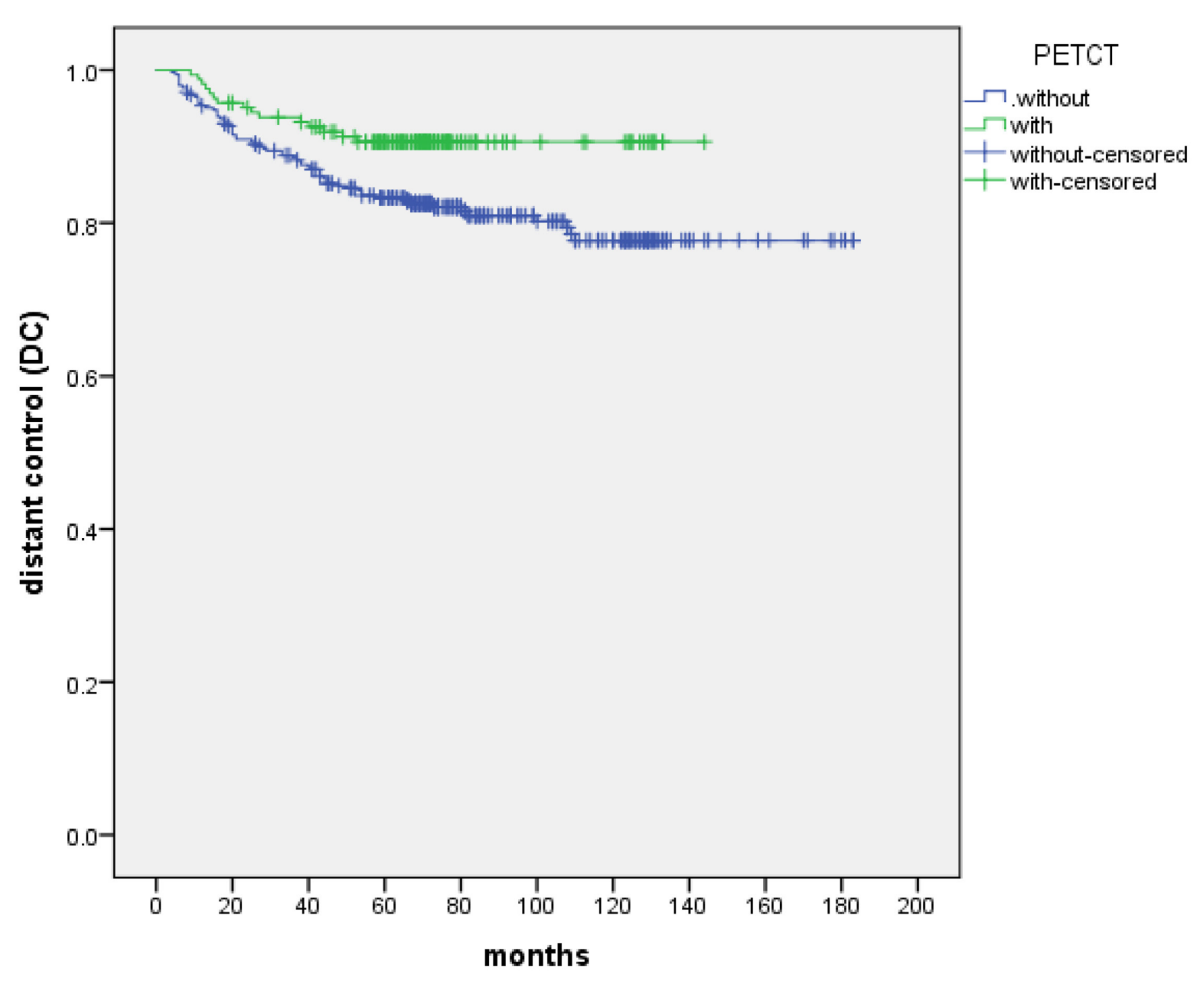
The DC between patients with the control and PET-CT group, respectively

The 5-year and 8-year DC were 85.6% and 83.8%, respectively. DC was not significantly different in the T stage (P=0.115) and the treatment (IC+IMRT+AC or IC+CCRT) of chemotherapy (P=0.532). N stage appeared to be a prognostic factor for DC (P=0.001). Patients with initially PET-CT-based staged showed improved 5-year and 8-year DC compared with the control group (90.6% vs. 83.3% and 90.6% vs. 81.0%, P=0.013) (Figure 2, Table 4).

### Local control and regional control

The 5-year and 8-year LC were 89.4% and 87.6%, respectively. T stage (P=0.000) was the factor that significantly influenced LC. Patients with T4 compared with those of T1-3 have worse LC. There was no significant difference in LC between the control and PET-CT group for T1, T2, T3, and T4 (P = 0.658, 0.725, 0.084, 0.207). The 5-year and 8-year RC were 91.5% and 89.9%, respectively. Patients with N2-3, compared with those of N0-1 have worse RC (5-year rates, 89.4% vs. 96.0%, 8-year rates, 86.9% vs. 96.0%, P=0.009). Initially PET-CT-based staged was not the factor that significantly influenced LC (P=0.494) and RC (P=0.618).

## Discussion

NPC is a malignant neoplasm of the epithelial tissue, which is sensitive to radiotherapy (RT) and chemotherapy and diagnosed as stage I-II for only 18.4%-25.4% at initial diagnosis 23-26. More than 70% of newly diagnosed NPC cases are classified as locoregionally advanced disease and CCRT plays an essential role in the treatment of NPC. The 5-year OS rates of stage III, IVA and IVB (AJCC 7th) patients were 79.1-86.0%%, 65.1-74.3% and 48.6-63.1% 8, 26-27. However, due to the high incidence of acute toxicity like oral mucositis, the tolerance of CCRT was unsatisfied and non-CCRT could also be considered as a treatment option for locoregionally advanced NPC 28-32. Wang P 28 showed that compared with CCRT in other literature, IC-RT-AC achieved similar long-term survivals. The 5-year OS rates for patients with stage III or IVA were 86.5% and 56.5%. Yang Z 30 prospectively evaluated that there was no significant difference (P>0.05) in OS between IC+CCRT (102 cases) and CCRT (102 cases) for locoregionally advanced NPC. In this study, we retrospectively evaluated the 5-year and 8-year OS rates of stage III and IVA were 88.2% and 78.3%, 79.8% and 65.9% (P=0.000), respectively. OS was not significantly different between IC+RT+AC and IC+CCRT (P=0.286).

The proportion of distant metastasis in NPC at initial diagnosis is 9.9%-14.8% 1-3. Initially PET-CT-based staged can detect potential distant metastasis and help with accurate staging and therapy compared with the routine examination, especially in N2-3 patients 33-35. A meta-analysis 33 of NPC found that the pooled sensitivity and specificity were 85.7% and 98.1% for PET/CT (1474 patients), and 38.0% and 97.6% for CWUs (1329 patients). In a study 34 of 300 cases NPC, 61 cases (20.3%) were found to have distant metastases. In comparing CWUs (chest radiography, abdominal ultrasonography, and skeletal scintigraphy) with PET-CT, PET-CT was found to be more effective and higher sensitivity (P < 0.001). Further analysis showed that PET-CT was more effective in detecting chest and bone metastases (P < 0.001), and similar to abdominal ultrasound in detecting hepatic metastases (P=0.127). The study concluded that PET-CT could replace CWUs in primary M staging of NPC.

The main failure pattern in NPC is distant metastasis, followed by nasopharyngeal and regional lymph node recurrence 5-9, 23-27. Distant metastases can occur in a single or multiple sites and be related to the N stage, with the bone, lung, and liver sorted by occurrence 36-37. Al Tamimi AS 36 analyzed the distribution of 709 FDG avid lesions of NPC detected by PET/CT, of which 357/709 (50.35%) were locoregional nodal metastases (115 retropharyngeal, 226 cervical, and 16 supraclavicular). There were 352/709 (49.65%) distant metastases, including 104/352 (29.55%) in the chest, 45/352 (12.78%) in the abdomen, 11/352 (3.13%) in the pelvis, and 192/352 (54.55%) in the bones. Wu 23 analyzed the treatment outcomes of 614 NPC without distant metastasis over 10 years: 151 cases (24.6%) were stage I-II, and 463 cases (75.4%) were stage III-IV. After a median follow-up of 112.7 months, 123 cases (20.0%) had distant metastases and the 5-year and 10-year DMFS were 97.7% and 97.7% (N0), 85.9% and 83.8% (N1), 73.0% and 70.0% (N2), 54.4% and 54.4% (N3), respectively. In our study, the control group underwent ECT as reported in the literature, the different was that chest CT was used instead of chest X-ray, and abdominal enhanced CT instead of abdominal ultrasound. The long-term survival results in our research showed that the 5-year and 8-year DC were 83.3%, 81.0% and 90.6%, 90.6% in the control and PET-CT groups, respectively (P=0.013).

NPC is prone to invade out of the nasopharyngeal cavity and metastasize to regional lymph nodes. Clarifying the extent of tumor invasion of NPC is crucial for confirming stage, RT planning, and prognosis. Enhanced MRI is recommended as the first-line examination for NPC due to the high soft tissue resolution and multiparametric imaging 38-39. Several studies have shown that nasopharynx enhanced MRI has advantages in detecting adjacent soft tissue involvement, skull base, intracranial invasion, retropharyngeal lymph nodes and so on 40-43. However, owing to the low resolution, PET-CT alone may overestimate or underestimate retropharyngeal lymph nodes. Ng 16 analyzed 111 cases of newly diagnosed NPC to investigate with PET/CT and conventional imaging. PET/CT showed a discrepancy with head-and-neck MRI in 36 (32.4%) according to T stage. Among the discordant cases, MRI was superior in demonstrating tumor involvement in the retropharyngeal nodes, skull base, parapharyngeal space, intracranial area, and sphenoid sinus while PET/CT was superior sensitivity and specificity in demonstrating neck nodal metastasis 44-46. Yang SS 44 analyzed the data of T3N1M0 NPC cases: Among the 269 cervical lymph nodes pathologically positive and 191 negative, 96.7% of positive and 75.9% of negative were correctly detected by PET/CT, while only 88.5% of positive and 70.7% of negative were correctly diagnosed by MRI (p<0.001). Patients who underwent both PET/CT and MRI had better OS, FFS, DMFS and LRRFS than those who underwent MRI alone (5-year OS, 95.7% vs. 90.4%, p<0.001; 5-year FFS, 85.7% vs. 71.7%, p<0.001; 5-year DMFS, 93.9% vs. 87.9%, p<0.001; 5-year LRRFS, 93.0% vs. 81.4%, p<0.001). In our study, all patients in both the control and PET-CT groups underwent nasopharynx enhanced MRI. The 5-year and 8-year LC rates of the control and PET-CT group were 88.6% and 91.1%, 87.0% and 88.8%, respectively (p=0.494). The 5-year and 8-year RC rates of the control and PET-CT group were 91.3% and 91.7%, 90.2% and 89.0%, respectively (p=0.618). The cost-effectiveness of PET-CT scans is a critical consideration. The primary benefit of PET-CT scans lies in their ability to detect diseases at an early stage, which can be crucial for effective treatment. This early detection can lead to more effective treatment plans and better patient prognoses. In addition, PET-CT is invaluable in the staging and monitoring of cancer treatments, as demonstrated by the ability to detect changes in metabolic activity that may not be apparent with other imaging techniques. Limitations of the study such as retrospective data, single center and lack of EBV-DNA should be considered in the future research.

## Conclusion

Patients with initially PET-CT-based staged showed improved long-term DC compared with the control group (chest CT, enhanced abdominal CT and ECT) for locoregionally advanced NPC with nasopharynx enhanced MRI before therapy. Initially PET-CT-based staged and nasopharynx enhanced MRI are recommended routinely to confirm stage and improve efficacy in locoregionally advanced NPC.

## Ethical approval

The study was approved by the Institutional Review Board of the Fudan University Shanghai Cancer Center in accordance with the Declaration of Helsinki.

## Figures and Tables

**Table 1:** Characteristics of patients

Characteristic		No. of patients	
		Control group	PET-CT group
Age	≤48	165	96
	>48	179	67
Gender	Female	102	42
	Male	242	121
KPS	70-80	194	64
	90-100	150	99
T stage	T1	24	15
	T2	72	48
	T3	139	64
	T4	109	36
N stage	N0	24	9
	N1	102	27
	N2	129	75
	N3	89	52
Clinical stage	III	158	80
	IVa	186	83
Treatment	IC+CCRT	58	57
	IC+RT+AC	286	106
Diagnosis year	2005-2014	252	59
	2015-2017	92	104
Chemotherapy	PF	115	40
	TPF	229	123

**Table 2:** All death causes of patients

	Whole group	Control group	PET-CT group
nasopharyngeal ± neck lymph nodes	32	22	10
neck lymph nodes	7	5	2
distant metastasis	46	37	9
distant metastasis + nasopharyngeal ± neck lymph nodes	8	7	1
distant metastasis+neck lymph nodes	8	6	2
massive hemorrhage	1	1	0
second tumor	6	4	2
others	19	17	2

**Table 3:** OS of different groups

		No. of patients	5-year (%)	8-year (%)	p
T stage	T1	6/39	92.3	86.2	0.001
	T2	24/120	85.8	78.1	
	T3	44/203	84.7	75.1	
	T4	53/145	75.6	59.9	
N stage	N0	7/33	81.6	74.2	0.412
	N1	33/129	87.5	71.6	
	N2	45/204	83.2	77.4	
	N3	42/141	78.7	67.5	
Stage	III	43/238	88.2	79.8	0.000
	IV	84/269	78.3	65.9	
Treatment	IC+IMRT+AC	108/392	82.9	71.2	0.286
	IC+CCRT	19/115	82.9	82.9	
Chemotherapy	PF	49/155	75.9	63.8	0.009
	TPF	78/352	86.0	76.0	
Pre-treatment examination	Control group	99/344	81.1	70.8	0.087
	PET-CT group	28/163	86.9	74.6	

**Table 4:** DC of different groups

		No. of patients	5-year (%)	8-year (%)	p
T stage	T1	6/39	84.6	84.6	0.115
	T2	14/120	89.0	87.9	
	T3	29/203	88.5	84.5	
	T4	31/145	78.8	78.8	
N stage	N0	1/33	96.6	96.6	0.001
	N1	16/129	89.6	88.1	
	N2	27/204	89.4	85.9	
	N3	36/141	74.0	74.0	
stage	III	23/238	93.2	89.3	0.000
	IV	57/269	78.9	78.9	
treatment	IC+IMRT+AC	65/392	85.4	83.2	0.532
	IC+CCRT	15/115	86.5	86.5	
Chemotherapy	PF	25/155	83.9	83.0	0.730
	TPF	55/352	86.4	84.2	
Pre-treatment examination	Control group	65/344	83.3	81.0	0.013
	PET-CT group	15/163	90.6	90.6	

## Abbreviations

ECT: emission computed tomography
NPC: nasopharyngeal carcinoma
OS: overall survival
DC: distant control
LC: local control
RC: regional control
PFS: progression-free survival
RFS: relapse-free survival
DMFS: distant metastasis-free survival
IC: induction chemotherapy
CCRT: concurrent chemotherapy
AC: adjuvant chemotherapy
RT: radiotherapy
